# Breaking the hard-sphere model with fluorite and antifluorite solid solutions

**DOI:** 10.1038/s41598-023-29326-0

**Published:** 2023-02-08

**Authors:** Romain Vauchy, Shun Hirooka, Masashi Watanabe, Masato Kato

**Affiliations:** 1 Plutonium Fuel Development Center, Japan Atomic Energy Agency, 4-33 Muramatsu, Tōkai-Mura, Ibaraki 319-1194 Japan; 2 Nuclear Plant Innovation Promotion Office, Japan Atomic Energy Agency, 4002 Narita-Cho, Ōarai-Machi, Ibaraki 311-1393 Japan

**Keywords:** Materials science, Materials chemistry

## Abstract

Using the hard-sphere model with the existing tabulated values of ionic radii to calculate the lattice parameters of minerals does not always match experimental data. An adaptation of this crystallographic model is proposed by considering the cations and anions as hard and soft close-packed spheres, respectively. We demonstrate the relevance of this “hybrid model” by combining Pauling’s first rule with experimental unit-cell parameters of fluorite and antifluorite-structured systems to revise the ionic radii of their constitutive species.

Ionic crystals are the alternate and periodic stacking of cations and anions forming a structural lattice by the balance between their attractive and repulsive forces. In the hard-sphere model, these ions are in contact and their radii are reported to depend on their oxidation state and coordination number (noted C.N.)^1^. Assessing these solids’ lattice parameters from the ionic radii of the constitutive species is paramount to understanding their defect chemistry and/or variations in their composition. Pauling proposed five empirical rules that these crystals shall respect to be stable^2^. The first stipulates that the coordination number of the cation depends on the ionic radii ratio r_cation_/r_anion_ and that the latter should range between specific values determined by geometry construction; otherwise, the structure is unstable, and the coordination changes. Shannon^3^ highlighted that using the tabulated ionic radii to model such materials’ lattice structures does not always match the experimental values and/or Pauling’s rules. Some studies also propose revised ionic radii^4^ that better agree with diffraction experiments; however, they do not coincide with the geometrical criteria.

Fluorites and antifluorites are critical ionic solids for a myriad of applications such as catalysts^5^, electroceramics^6^, or even nuclear fuels^7^, and thanks to their high symmetry, their geometry is simple to model (Fig. 1). Their lattice parameter $a$ can be calculated using Eq. 1 from the body diagonal of the lattice (cube) and the ionic radii $r$ of the constitutive species.

1 $$a = \frac{4}{\sqrt 3 } \times \left( {r_{cation} + r_{anion} } \right)$$

**Figure 1:**
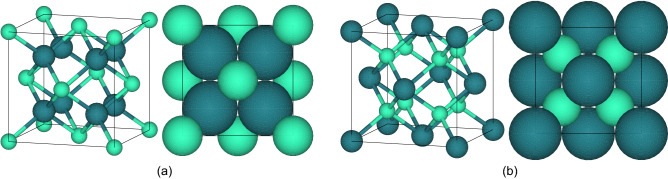
Unit-cell drawings of ideal (**a**) fluorite and (**b**) antifluorite structures and their respective projections (space-filling spheres) along the (100) plane. The small ocean-mint and large petrol-blue spheres represent the cations and anions, respectively.

The cations and anions are in 8- and fourfold coordination in fluorites, respectively. The structure is stable if 0.732 ≤ r_cation_/r_anion_ ≤ 1.000^8^. Since antifluorite is the fluorite’s antistructure, these ions’ positions are permuted and the crystallographic arrangement is stable if 0.225 ≤ r_cation_/r_anion_ ≤ 0.414^8^. These structures were widely studied in the past century and experimental lattice parameters are numerous. At the light of these crystals, we break the hard-sphere model by demonstrating that anions are soft because their ionic radius not only depends on their charge and coordination number but also on the nature (thus size) of the closest neighbors.

## Ideal fluorites & antifluorites

Table 1 shows Shannon’s^1^ ionic radii of the constitutive species of some selected important compounds for their use in various fields (fluorites: ZrO_2_, TbO_2_, HfO_2_, CeO_2_, ThO_2_, UO_2_, NpO_2_, PuO_2_, AmO_2_, CmO_2_, BkO_2_, CfO_2_, CaF_2_, SrF_2_, BaF_2_, EuF_2_, and PbF_2_; antifluorites: Na_2_O, Li_2_O, and K_2_O). The associated r_cation_/r_anion_ ratios, compared to the stability criteria of Pauling’s first rule, and their experimental lattice parameters at room temperature obtained using diffraction methods are also listed.

**Table 1:** Calculated r_cation_/r_anion_ ratios from Shannon’s ionic radii^1^ of selected fluorite and antifluorite-structured compounds and their experimental lattice parameters at room temperature determined using X-ray diffraction.

Structure	Compound	Ionic species	C.N	Ionic radius (Å)^1^	r_cation_/r_anion_	Lattice parameter (Å) at 298 K	Pauling’s electronegativity χ^9^	χ_anion_ − χ_cation_
Fluorite	–	O(–II)	4	1.38	–	–	3.44	–
	ZrO_2_	Zr(IV)	8	0.84	**⁓ 0.609**	5.135(5)^10^	1.33	2.11
	TbO_2_	Tb(IV)	8	0.88	**⁓ 0.638**	5.213(2)^11^	1.20	2.24
	HfO_2_	Hf(IV)	8	0.83	**⁓ 0.601**	5.115^12^	1.30	2.14
	CeO_2_	Ce(IV)	8	0.97	**⁓ 0.703**	5.411(1)^13^	1.12	2.32
	ThO_2_	Th(IV)	8	1.05	*⁓ 0.761*	5.5971(5)^14^	1.30	2.14
	UO_2_	U(IV)	8	1.00	**⁓ 0.723**	5.47127(8)^15^	1.38	2.06
	NpO_2_	Np(IV)	8	0.98	**⁓ 0.710**	5.4336(5)^14^	1.36	2.08
	PuO_2_	Pu(IV)	8	0.96	**⁓ 0.696**	5.3954(5)^16^	1.28	2.16
	AmO_2_	Am(IV)	8	0.95	**⁓ 0.688**	5.3755(5)^16^	1.13	2.31
	CmO_2_	Cm(IV)	8	0.95	**⁓ 0.688**	5.3598(4)^17^	1.28	2.16
	BkO_2_	Bk(IV)	8	0.93	**⁓ 0.674**	5.3304(8)^18^	1.30	2.14
	CfO_2_	Cf(IV)	8	0.92	**⁓ 0.667**	5.310(2)^19^	1.30	2.14
	-	F(–I)	4	1.31	–	–	3.98	–
	CaF_2_	Ca(II)	8	1.12	*⁓ 0.855*	5.4779(4)^20^	1.00	2.98
	SrF_2_	Sr(II)	8	1.26	*⁓ 0.962*	5.8771(7)^20^	0.95	3.03
	BaF_2_	Ba(II)	8	1.42	⁓ 1.084*	6.200(1)^21^	0.89	3.09
	EuF_2_	Eu(II)	8	1.25	*⁓ 0.954*	5.808^22^	1.20	2.78
	PbF_2_	Pb(II)	8	1.29	*⁓ 0.985*	5.940(1)^23^	1.87	2.11
Antifluorite	-	O(–II)	8	1.42	–	–	3.44	–
	Na_2_O	Na(I)	4	0.99	**⁓ 0.697**	5.544(2)^24^	0.93	2.51
	Li_2_O	Li(I)	4	0.59	**⁓ 0.415**	4.6117(5)^25^	0.98	2.46
	K_2_O	K(I)	4	1.37	**⁓ 0.993**	6.436^26^	0.82	2.62

*BaF_2_ is a peculiar example as the cation is larger than the anion (r_cation_/r_anion_ > 1). From a geometrical stability point of view, it shall be considered as an antifluorite as the smaller ion is in fourfold coordination instead of 8.

The ratios highlighted in italic and bold correspond to the structures that respect and violate Pauling’s first rule, respectively. Pauling’s electronegativity χ^9^ values and χ_anion_ − χ_cation_ are also listed.

First of all, the difference in Pauling’s electronegativity χ_anion_ − χ_cation_ in the selected compounds is larger than the minimum value of ⁓ 1.7 that defines the ionic solids^8,9^. Indeed, Δχ ranges between 2.06 (UO_2_) and 3.09 (BaF_2_). Discussing the ionic radii of the constitutive species is then legitimate and the high ionicity of the bonds allows approximating ions as spherical entities.

The compounds highlighted in bold in Table 1 should not be stable if the r_cation_/r_anion_ lower limit of Pauling’s first rule is respected or if Shannon's ionic radii are correct. To fulfill the geometric stability criteria and to match the experimental unit-cell measurements of these ionic crystals, we consider the anions as soft spheres with an effective radius varying as a function of the cation’s nature by deriving Eq. (1) in Eq. (2).

2 $$r_{anion} = a \times \frac{\sqrt 3 }{4}{-}r_{cation}$$

In the peculiar case of the fluorites highlighted in bold in Table 1, the r_cation_/r_anion_ ratio is smaller than the lower stability limit, so cations and anions are not in contact in this configuration. The unit-cell is a close-packed arrangement of anions and can be calculated using Eq. 3.

3 $$a = 4 \times r_{anion}$$

If a fixed anionic radius is considered, these compounds’ lattice parameters will also be, geometrically, independent of the nature of the cations that partly fill the interstices of the structure (Fig. 1). However, this is not verified experimentally, as evidenced by the variations in the lattice parameters observed when the cation is changed (Table 1). The cations and anions shall be, at least, in contact one another^2^. Thus, from the accurate experimental lattice parameters, applying Eq. (3) to UO_2_ and PuO_2_ gives two oxygen radii of 1.368 and 1.349 Å, respectively. Implementing these values in Eq. (2) allows determining the revised cation radii giving 1.001 and 0.987 Å for U(IV) and Pu(IV) in 8-coordination, respectively. Similarly, the constitutive species’ ionic radii of the bold fluorite compounds in Table 1 were re-evaluated (Table 3 in supplementary materials).

**Figure 2:**
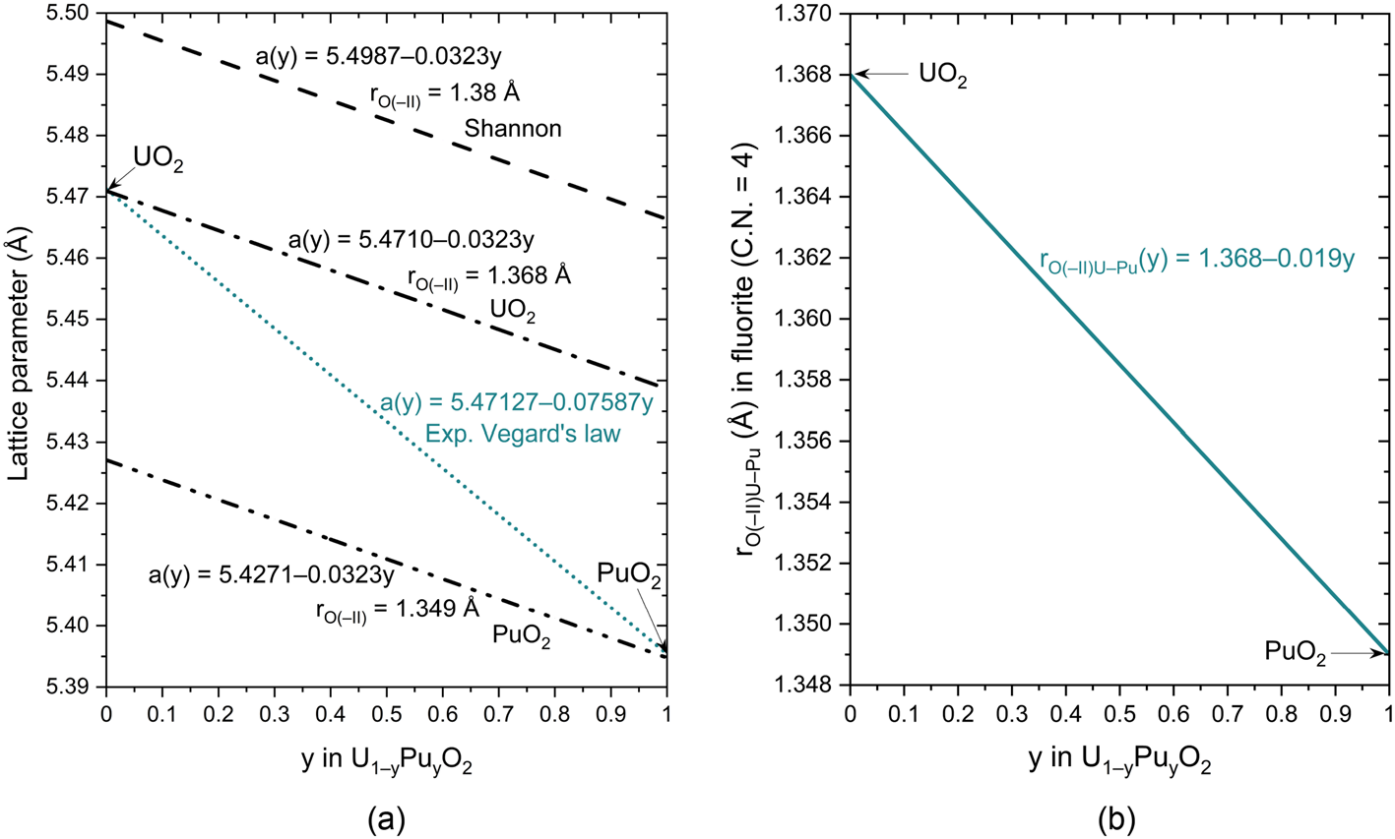
Variations in (**a**) experimental lattice parameter of U_1−y_Pu_y_O_2_ at room temperature as a function of y (petrol-blue dotted line) compared with calculations (Eq. 1) from the hard-sphere model with three oxygen ionic radii (black dashed lines) and (**b**) the ionic radius of oxygen as a function of y, noted r_O(–II)U–Pu_.

UO_2_ and PuO_2_ are known to form a solid solution of U_1−y_Pu_y_O_2_, i.e., U and Pu can be substituted in their mutual lattice. When the two oxygen ionic radii are individually used to plot the theoretical Vegard’s law between UO_2_ and PuO_2_, no value matches the experimental variations in the fluorite structure’s lattice parameter as a function of the Pu content, *y* (Fig. 2a). As the commuted species, U and Pu do not have the same ionic radii, the crystal’s geometry is locally deformed. Since the ions are considered contacted spheres, the cation–anion distance will change when incorporating the doping species in the host lattice. To do so, and by considering the anions as a close-pack arrangement, the r_anion_ will vary proportionally to the incorporation of the doping cation within the lattice. Figure 2b plots the variations in the oxygen ion size as a function of the plutonium concentration in the U_1−y_Pu_y_O_2_ solid solution from the linear regression between UO_2_ and PuO_2_. This model allows reproducing of the experimental lattice parameters of U_1−y_Pu_y_O_2_ and matches the geometrical criteria established by Pauling.

As antifluorites are the antistructure of fluorites, the same method can be used to estimate their solid solutions’ lattice parameters from the pure constitutive poles.

If the coordination number and the oxidation state of the considered cations do not change, this method could be used for any ionic crystal and predict the lattice parameters of solid solutions not yet investigated experimentally, as long as the difference in cations’ electronegativity, Δχ_M_, is small. Indeed, a large Δχ_M_ results in a disordered structure due to the resulting different Cation–Anion bond distances. Ultimately, the gradual incorporation of a dopant element within the host lattice changes the local coordination number of one of the cations, even if they have identical oxidation sates and comparable ionic radii^27^.

## Incorporating aliovalent cations

Doping is widely used for tailoring a given material’s properties (optical, electrical, redox). Aliovalent atoms are frequently used as dopants to boost the target properties. Also, some cations can have various oxidation states within the same structure, resulting in a deviation from stoichiometry. Due to their exceptional aptitude to form solid solutions, fluorites and antifluorites are often doped, even with aliovalent cations, and can likewise be subjected to dramatic variations in stoichiometry^7,16^ and/or complex charge compensation processes^28^. Such intrusive atoms generate lattice distortions and/or crystal defects, and the ratio of the ionic radii of the two permuted atoms will be considered to evaluate the effect of such a dopant on the host structure’s lattice parameters.

However, using our method in such materials might be problematic because one of the pure poles might crystallize in a different structure than the host lattice. For instance, in Nd-doped UO_2_ fluorite, neodymium is trivalent, and its oxide form is Nd_2_O_3_ (either cubic or haxagonal^29^); thus, a direct application of our method cannot estimate the variations in the oxygen radius with the Nd concentration in U_1−y_Nd_y_O_2_. Therefore, we propose an alternate method that compares the fluorite/nonfluorite pseudo-binary system to a known fluorite/fluorite couple. Hence, our UO_2_/Nd_2_O_3_ example can be paralleled to the well-known UO_2_/PuO_2_ system by comparing the sizes of Pu(IV) and Nd(III) ions in eightfold coordination. Also, doping UO_2_ with trivalent neodymium should be balanced by a partial oxidation of uranium to its pentavalent state in the same proportions as the Nd incorporation^30^. Thus, the size of U(V) will be considered. Table 2 presents the ionic radii of the constitutive cations (C.N. = 8) and the associated r_cation_/r_Pu(IV)_ ratios.

**Table 2:** Ionic radii ratios r_cation_/r_Pu(IV)_ of the doping species in fluorite Nd- and Pu-doped UO_2_.

Ionic species	Ionic radius r (Å)	r_cation_/r_Pu(IV)_ (%)
U(V)	0.88^31^	− 10.84
U(IV)	⁓ 1.001^32^*	+ 1.42
Pu(IV)	⁓ 0.987^32^**	–
Nd(III)	1.109^1^	+ 12.36

*Calculated with ${\mathrm{a}}_{{\mathrm{UO}}_{2}}$ equal to 5.47127(8) Å at 298 K^15^. **Calculated with ${\mathrm{a}}_{{\mathrm{PuO}}_{2}}$ equal to 5.3957(5) Å at 298 K^16^.

**Figure 3:**
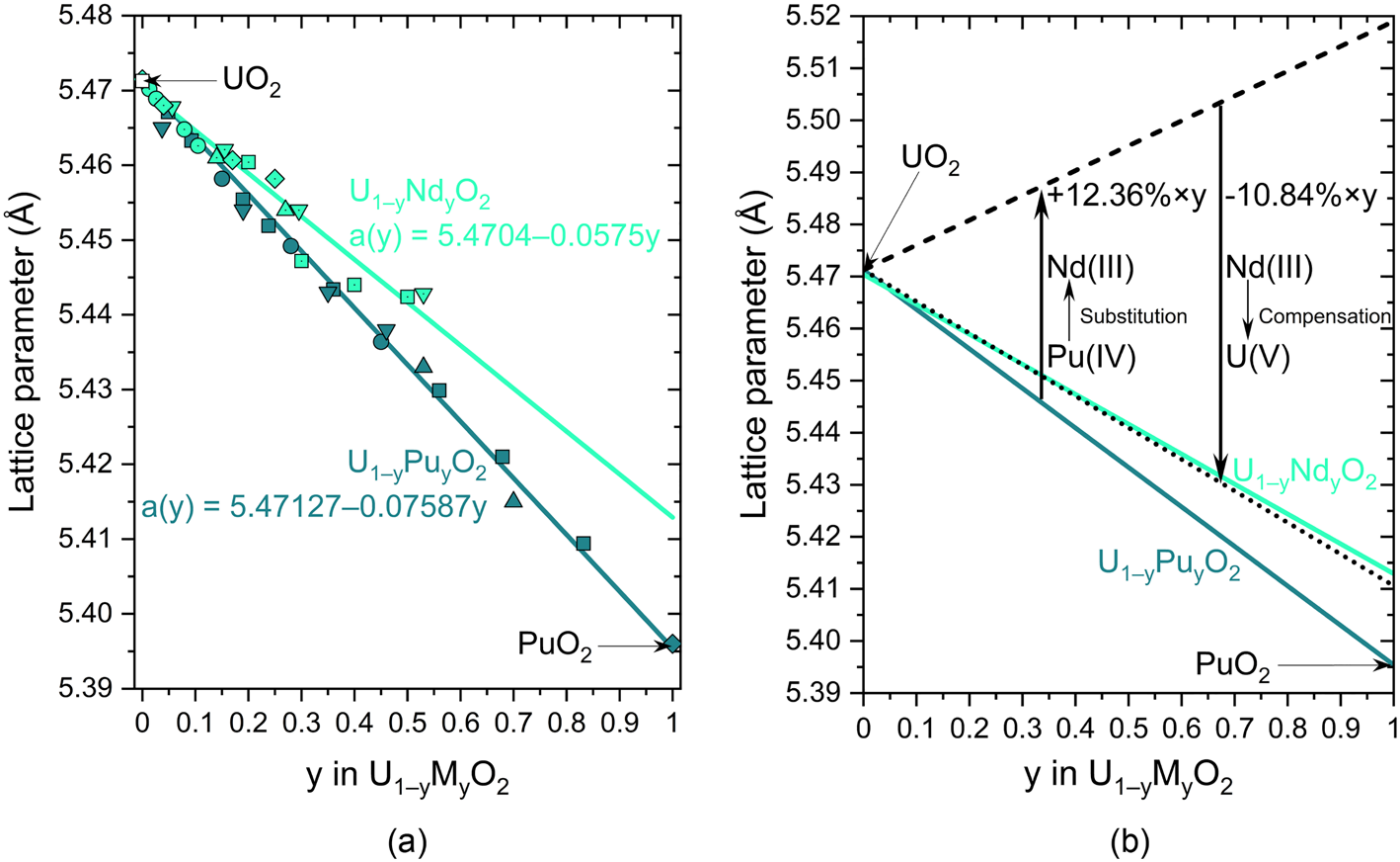
(**a**) Variations in the experimental lattice parameters of U_1−y_M_y_O_2_ at room temperature as a function of *y* with M=Pu or Nd. The symbols correspond to the experimental data at room temperature, and the lines to their linear regression as a function of *y* (Vegard’s law). (**b**) Comparison of experimental (solid lines) and calculated lattice parameters from the tabulated ionic radii without (dashed line) and with (dotted line) the Nd(III)/U(V) compensation mechanism. N.B. the fit of ${a}_{{U}_{1-y}{Nd}_{y}{O}_{2}}\left(y\right)$ was extrapolated to *y* = 1 for the discussion, even if the fluorite structure is not maintained at high Nd concentrations.

Figure 3a plots the variations in the experimental lattice parameters of U_1−y_M_y_O_2_ as a function of the dopant content y with M = Pu^16,33–36^ or Nd^30,31,37–39^, and (b) represents the calculated theoretical lattice parameters of U_1−y_Nd_y_O_2_ from the ionic radii ratios of Table 2.

The exquisite agreement between the experimental and calculated lattice parameters of U_1−y_Nd_y_O_2_ represented in Fig. 3b confirms that: (1) the theoretical crystallography calculations verify that the Nd(III)/U(V) charge compensation model in U_1−y_Nd_y_O_2_ is correct, and (2) our geometrical hybrid model based on the parallelism between the considered fluorite/nonfluorite system to a known fluorite/fluorite one is successful. However, one may keep in mind that the disorder generated by the incorporation of a dopant cation (aliovalent or not) within the lattice may affect the unit-cell parameters, inducing deviations from the pseudo-Vegard’s law^40^.

Similarly, the investigation of higher-order systems is possible. For example, stoichiometric U_1−y_M_y_O_2_ with M = Pu + Am is a complex system where americium can take a pure tetravalent oxidation state, a mixed Am(III)/Am(IV) valence, or be purely trivalent depending on its concentration and distribution homogeneity^41^. Likewise, Am(III) in uranium–plutonium–americium mixed oxides is balanced by U(V)^28^, but a clear trend regarding the Am(III)/Am ratio remains to be determined. In pure americium dioxide, the r_cation_/r_anion_ is smaller than Pauling’s geometrical limit (Table 1), and the anions should touch and form a close-pack arrangement. Since the lattice parameter of AmO_2_ at room temperature is 5.3755(5) Å^16^, the ionic radius of O(–II) in this compound is 1.344 Å, and gives, using Eq. (2), the revised cation radius of r_Am(IV)_ = 0.984 Å (for C.N. = 8). The ionic radius of Am(III) in eightfold coordination is taken from Cross^42^ and equal to 1.108 Å.

From these ionic radii and by using the same method as above, the variations in the lattice parameters of U_1−y_{Pu_1−α_[Am(IV)_1−β_Am(III)_β_]_α_}_y_O_2_ as a function of plutonium, americium, and Am(III)/Am contents can be calculated using Eq. (4).

4 $$a_{{U_{{1{-}y}} \left\{ {Pu_{{1{-}\alpha }} \left[ {Am\left( {IV} \right)_{{1{-}\beta }} Am\left( {III} \right)_{\beta } } \right]_{\alpha } } \right\}_{y} O_{2} }} \left( {y,\alpha ,\beta } \right) = \frac{4}{\sqrt 3 } \times \left[ {\left( {1{-}y{-}\beta \cdot \alpha \cdot y} \right) \times r_{{U\left( {IV} \right)}} + \left( {1{-}\alpha } \right) \times y \times r_{{Pu\left( {IV} \right)}} + \left( {1{-}\beta } \right) \times \alpha \times y \times r_{{Am\left( {IV} \right)}} + \beta \times \alpha \times y \times r_{{Am\left( {III} \right)}} + \beta \times \alpha \times y \times r_{U\left( V \right)} + r_{{O\left( {{-}II} \right)U - Pu - Am}} \left( {y,\alpha ,\beta } \right)} \right]$$

with the oxygen ionic radius $r_{{O\left( {{-}II} \right)U - Pu - Am}} \left( {y,\alpha ,\beta } \right)$ calculated in the same manner as for the Nd-doped UO_2_.

## Deviation from stoichiometry in fluorite-structured oxides

In addition to their ability to form solid solutions, the fluorite structure (oxides) can accommodate large deviations from stoichiometry^7,16,43^. We enlarged our hybrid model to nonstoichiometric materials. In oxygen-hypostoichiometric fluorite-structured dioxides (Oxygen/Metal < 2), Kim^44^ and Chatzichristodoulou^45^ have envisaged a flexible oxygen vacancy size but considered a fixed ionic oxygen radius. Since hypostoichiometry corresponds to removing anions from the crystal, the associated reduced cations’ coordination number should, theoretically, be modified likewise (Fig. 1). However, due to the Born–Haber energy, the lattice does not collapse, therefore, we considered the oxygen vacancies as spherical entities instead of empty voids. The coordination number of the cations remains unchanged and equal to 8.

For discussion, we examined the fluorite-structured hypostoichiometric uranium–plutonium mixed oxides U_1−y_Pu_y_O_2−x_ for which an empirical relation between the experimental lattice parameters (in Å) and deviations from stoichiometry at room temperature^31,46^ exists. It is updated in Eq. (5) from the recent measurements of the accurate lattice parameters of UO_2_^15^ and PuO_2_^16^.

5 $$a_{{U_{{1{-}y}} Pu_{y} O_{{2{-}x}} }} \left( {y,x} \right) = a_{{UO_{2} }} + \frac{{a_{{PuO_{2} }} - a_{{UO_{2} }} }}{1 - 0} \times y + 0.32 \times x = 5.47127 - 0.07587 \times y + 0.32 \times x$$

**Figure 4:**
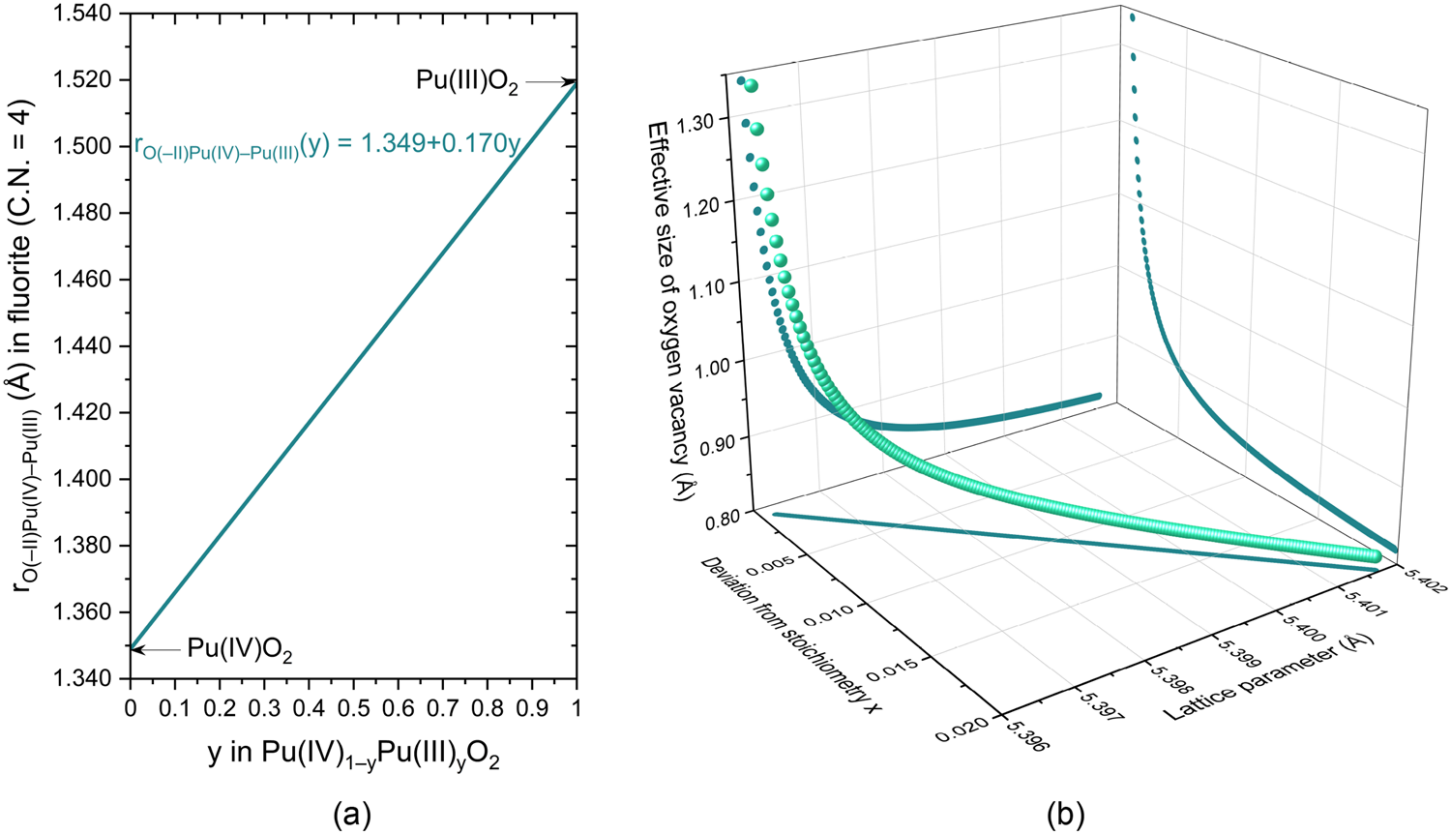
Variations in (**a**) the ionic radius of oxygen, noted r_O(–II)Pu(IV)–Pu(III)_, as a function of y in the hypothetical Pu(IV)_1−y_Pu(III)_y_O_2_ dioxide and (**b**) in the effective size of the oxygen vacancy as a function of the deviation from stoichiometry and the lattice parameter of PuO_2−x_ at room temperature. N.B. *x* was only set up to 0.02 because PuO_1.98_ is the more reduced composition plutonium dioxide can reach without changing its structure at room temperature, i.e., without forming a second phase.

In U_1−y_Pu_y_O_2−x_, Pu(IV) can be partially reduced to Pu(III) and is solely responsible for the hypostoichiometry in the mixed oxide below ⁓ 1700 K. Due to the solid’s electroneutrality, the deviation from stoichiometry *x* is directly correlated to the valence of the constitutive cations. If we simplify the system by focusing on the PuO_2−x_ dioxide (so U_1−y_Pu_y_O_2−x_ with y = 1) and considering that the oxygen vacancies are doubly charged and balanced by two Pu(III), PuO_2−x_ can be defined as Pu(IV)_1−2x_Pu(III)_2x_O_2−x_. Since tetra- and trivalent Pu have different ionic radii in the eightfold coordination, PuO_2−x_ will be treated as a solid solution of the hypothetical Pu(IV)O_2_–Pu(III)O_2_ system (even if the second end member has no physical meaning). Before estimating the oxygen vacancy’s effective size in PuO_2−x_, the oxygen ionic radius r_O(–II)_ must be determined. Therefore, the ionic radius of Pu(III) in the eightfold coordination, taken from Cross^42^ and equal to 1.112 Å, is implanted in the lower boundary of Pauling’s first rule, 0.732 = r_cation_/r_anion_ (for C.N. = 8), to calculate the anion’s radius in the hypothetical Pu(III)O_2_ dioxide. Figure 4a plots the variations in the ionic radius of oxygen as a function of y in Pu(IV)_1−y_Pu(III)_y_O_2_. Equation (2) is then derived to obtain Eq. (6).

6 $$r_{{V_{{O\left( { - II} \right)PuO_{{2{-}x}} }} }} \left( x \right) = \frac{{a_{{PuO_{{2{-}x}} }} \left( x \right) \times \sqrt 3 }}{2 \times x} + \frac{{4 \times x{-}2}}{x} \times r_{{Pu\left( {IV} \right)}} {-}4 \times r_{{Pu\left( {III} \right)}} - \frac{{2{-}x}}{x} \times r_{{O\left( {{-}II} \right)Pu\left( {IV} \right) - Pu\left( {III} \right)}}$$

where $r_{{V_{{O\left( {{-}II} \right)PuO_{2 - x} }} }}$ and $r_{{O\left( {{-}II} \right)Pu\left( {IV} \right) - Pu\left( {III} \right)}}$ (from Fig. 4a) are the effective sizes of the anion vacancy and the ionic radius of oxygen in Pu(IV)_1−y_Pu(III)_y_O_2_, respectively. Figure 4b shows the variations in $r_{{V_{{O\left( {{-}II} \right)PuO_{2 - x} }} }}$ as a function of the deviation from stoichiometry *x* and the lattice parameter of PuO_2–x_ at room temperature, from combining Eqs. (5) and (6).

These results confirm that the oxygen, vacancy’s effective size is not constant, likewise the ionic radius of oxygen, and it depends on the dopant’s concentration, here Pu(III), and rapidly decreases and stabilizes with the increasing deviation from stoichiometry.

We believe this method can be used for more complex systems, including solid solutions and/or compounds with aliovalent cations, such as plutonium in PuO_2−x_.

## Conclusions

This innovative method cleaves with the globally adopted model of ionic radii only depending on the constitutive species’ oxidation state and coordination number. Using simple structures, such as fluorites and antifluorites, we break the hard-sphere model by demonstrating that the anions are soft because their radius varies with the nature (size) of the surrounding cations. This hybrid model does not violate Pauling’s first rule and can predict the lattice parameters of new fluorite and antifluorite-structured solid solutions if the pure poles are known, as long as the host and dopant metal atoms have similar electronegativities. Finally, this geometrical method’s transposition is possible to investigate the deviations from stoichiometry. This result confirms that, in hypostoichiometry, the oxygen vacancy’s effective size will not be considered constant and depends on the doping species’ nature and concentration. We also reasonably envisage that this method could be used for any ionic crystal.

## Supplementary Information

Supplementary Table S1.

## Data Availability

All data generated or analyzed during this study are included in this published article and its supplementary information files.
