# Publisher Correction: Breaking the hard-sphere model with fluorite and antifluorite solid solutions

**DOI:** 10.1038/s41598-023-33087-1

**Published:** 2023-04-21

**Authors:** Romain Vauchy, Shun Hirooka, Masashi Watanabe, Masato Kato

**Affiliations:** 1 Plutonium Fuel Development Center, Japan Atomic Energy Agency, 4‑33 Muramatsu, Tōkai‑Mura, Ibaraki 319‑1194 Japan; 2 Nuclear Plant Innovation Promotion Office, Japan Atomic Energy Agency, 4002 Narita‑Cho, Ōarai‑Machi, Ibaraki 311‑1393 Japan

Correction to: *Scientific Reports* 10.1038/s41598-023-29326-0, published online 08 February 2023

The original version of this Article contained an error in Table 1, where several values were incorrectly italicised in column r_cation_/r_anion_ ratios. The correct and incorrect values appear below.

Incorrect:

**Table:** 

Structure	r_cation_/r_anion_
Fluorite	–
	**~ 0.609**
	**~ 0.638**
	**~ 0.601**
	**~ 0.703**
	~ 0.761
	**~ 0.723**
	**~ 0.710**
	**~ 0.696**
	**~ 0.688**
	**~ 0.674**
	**~ 0.667**
	–
	~ 0.855
	~ 0.962
	~ 1.084*
	~ 0.954
	~ 0.985
Antifluorite	–
	**~ 0.697**
	**~ 0.415**
	**~ 0.993**

Correct:

**Table:** 

Structure	r_cation_/r_anion_
Fluorite	–
	**~ 0.609**
	**~ 0.638**
	**~ 0.601**
	**~ 0.703**
	*~ 0.761*
	**~ 0.723**
	**~ 0.710**
	**~ 0.696**
	**~ 0.688**
	**~ 0.674**
	**~ 0.667**
	–
	*~ 0.855*
	*~ 0.962*
	~ 1.084*
	*~ 0.954*
	*~ 0.985*
Antifluorite	–
	**~ 0.697**
	**~ 0.415**
	**~ 0.993**

As a result, in section Ideal fluorites & antifluorites,

“The compounds highlighted in italic and bold in Table 1 should not be stable if the r_cation_/r_anion_ lower limit of Pauling’s first rule is respected or if Shannon’s ionic radii are correct.”

now reads:

“The compounds highlighted in bold in Table 1 should not be stable if the r_cation_/r_anion_ lower limit of Pauling’s first rule is respected or if Shannon’s ionic radii are correct.”

Also,

In the peculiar case of the fluorites highlighted in italic and bold in Table 1, the r_cation_/r_anion_ ratio is smaller than the lower stability limit, so cations and anions are not in contact in this configuration.

now reads:

In the peculiar case of the fluorites highlighted in bold in Table 1, the r_cation_/r_anion_ ratio is smaller than the lower stability limit, so cations and anions are not in contact in this configuration.

And,

Similarly, the constitutive species’ ionic radii of the italic and bold fluorite compounds in Table 1 were re-evaluated (Table 3 in supplementary materials).

now reads:

Similarly, the constitutive species’ ionic radii of the bold fluorite compounds in Table 1 were re-evaluated (Table 3 in supplementary materials).

The original Article has been corrected.

