# Supplementary material for: Breaking the hard-sphere model with fluorite and antifluorite solid solutions
Source: Sci Rep. 2023 Feb 8;13:2217. doi: 10.1038/s41598-023-29326-0 (PMC9908875; doi:10.1038/s41598-023-29326-0)
Supplement: Supplementary file 1 — Supplementary Table S1. [file 41598_2023_29326_MOESM1_ESM.docx]

**Supplementary materials**

Table 3 gives the revised anionic and cationic radii in the selected compounds.

Table 3. Anionic and revised cationic radii (Å) in selected fluorite-structured compounds

| Compound | Anion | | | Cation | | |
| --- | --- | --- | --- | --- | --- | --- |
|  | C.N. | Species | Revised ionic radius (Å) | C.N. | Species | Revised ionic radius (Å) |
| ZrO_2_ | 4 | O(–II) | 1.284 | 8 | Zr(IV) | 0.940 |
| TbO_2_ | 4 | O(–II) | 1.303 | 8 | Tb(IV) | 0.954 |
| HfO_2_ | 4 | O(–II) | 1.279 | 8 | Hf(IV) | 0.936 |
| CeO_2_ | 4 | O(–II) | 1.353 | 8 | Ce(IV) | 0.990 |
| UO_2_ | 4 | O(–II) | 1.368 | 8 | U(IV) | 1.001 |
| NpO_2_ | 4 | O(–II) | 1.358 | 8 | Np(IV) | 0.994 |
| PuO_2_ | 4 | O(–II) | 1.349 | 8 | Pu(IV) | 0.987 |
| AmO_2_ | 4 | O(–II) | 1.344 | 8 | Am(IV) | 0.984 |
| CmO_2_ | 4 | O(–II) | 1.340 | 8 | Cm(IV) | 0.981 |
| BkO_2_ | 4 | O(–II) | 1.333 | 8 | Bk(IV) | 0.976 |
| CfO_2_ | 4 | O(–II) | 1.328 | 8 | Cf(IV) | 0.972 |
